# Towards an Efficient Identification Process for Large-Scale RFID Systems †

**DOI:** 10.3390/s18072350

**Published:** 2018-07-19

**Authors:** Leonardo Sanchez, Victor Ramos

**Affiliations:** 1 Department of Systems, Universidad Autónoma Metropolitana–Azcapotzalco, CDMX 02200, Mexico; ldsm@correo.azc.uam.mx; 2 Department of Electrical Engineering, Universidad Autónoma Metropolitana–Iztapalapa, CDMX 09340, Mexico

**Keywords:** distributed identification, RFID systems, EPC-Gen2

## Abstract

Radio Frequency Identification (RFID) is one of the most widely used wireless communications technologies nowadays. Among the numerous processes executed within an RFID system, the identification processis the most important one. There have been several proposals to efficiently execute such a mechanism, which are based on the use of an RFID identification method. Besides, one of the most studied scenarios comprises one reader and a set of RFID tags, which we call the centralized approach. Recent work shows that executing the identification process in a distributed or parallel way may be of great benefit for applications with high requirements on time and resources usage, i.e., applications where the time required to execute the identification process needs to be low. In this paper, we focus is on large RFID systems and compare two identification mechanisms, one based on the centralized approach and the other based on the distributed approach. Our aim is to find the advantages and disadvantages of each approach for general RFID scenarios. We observe that the distributed approach is very promising compared to the traditional approach since considerable improvements are found in identification delay, and also the implementation costs would be highly reduced.

## 1. Introduction

As the number of objects connected to the Internet increases, the need for efficient identification mechanisms increases as well. RFID is one of the most widely used auto-ID technologies in the industry and research. The main attraction of RFID over other wireless technologies, such as bar codes, is its ability to identify objects simultaneously in a wireless way [1]. Another important aspect of RFID technology is its implementation cost. Passive RFID technology is very cheap compared to other wireless technologies, such as wireless sensor networks. Thus, RFID is being used in a wide variety of applications, from public transportation to tracking and monitoring objects.

An RFID system is a collection of devices that provides information related to a set of interest objects in a given area. The interest objects can be persons, animals or items that are related to the application for which the RFID system is designed. The RFID system is composed of an RFID network and a storage system. The RFID network is the core of the RFID system, it contains the information related to the interest objects. There is at least one reader and several RFID tags in an RFID network. The number of RFID readers depends on the features of the RFID reader, the application purpose, and the size of the interest area. Each reader defines a coverage area or interrogation zone according to its physical capabilities. Each tag is attached to one of the objects within the interest area. Therefore, the number of tags is equal to the number of objects within the interest area. Each tag has a unique ID; it may be passive or active. Passive tags do not account with an internal power source, they are rather powered by the electromagnetic waves transmitted by the reader. Also, passive tags have reduced storage and processing capabilities, they cannot sense the medium or start a communication with another device, either reader or tag. Thus, passive tags are very cheap compared to active tags, that is why they are preferred in industry and research. Moreover, the storage system is composed by a back end system and a middleware. The latter is charged with integrating the information collected by the set of RFID readers in order to store it on the back end system.

RFID networks were conceived with a centralized approach. In a typical RFID network there is only one reader and a set of tags within the reader’s coverage area. An RFID network can execute many processes, like tag-set estimation, monitoring a set of tags, and tag identification (also known as the identification process). Therefore, two or more RFID networks can be integrated into a big network, with more than one RFID reader on the same region in order to completely cover an interest zone; this is known as a Multi-Reader Scheme (MR-Scheme). This forces these networks to work in a cooperative way to execute any of the processes just mentioned. Among these processes, the identification process is the most important one since it is executed at least once on every RFID system. The identification process consists in gathering the ID of every tag within the interest area. As the number of tags increases, it is more difficult to execute the identification process. Thus, due to the impact of the identification process on RFID systems, the research community has made many efforts in order to improve its performance. These efforts range from enhancing the network devices to designing efficient communication protocols [2]. In recent years, there has been intensive research work seeking to replace the traditional centralized architecture of RFID networks with distributed or parallel approaches; thus, this idea introduces a new paradigm for RFID networks.

Since the RFID reader is the source of information requests as well as the destination of tags transmissions, the main idea is to reduce the workload of the reader. Hence, the new paradigm mainly proposes to separate the functions executed by the reader, transmission and reception of information, in two spatially separated devices with specific functions. Thus, the key idea is to delegate the reception of information to a new device, while the reader only executes information requests. Since this new approach involves more devices than the traditional centralized approach, these types of systems are renamed as augmented RFID systems.

The first attempts to distribute the identification process did not include a new device in the RFID network. The identification process was executed in a distributed way by using particular anticollision protocols [3,4,5]. Therefore, in literature, there are two important contributions that are pioneers in this research direction. In [6], Ali and Hassanein propose an augmented RFID system with multiple receiving devices called fielders. A fielder is a device similar to a node in a sensor network with limited resources and a limited lifetime in terms of energy. Among the functions executed by a fielder there is the interaction with the reader to create micro-zones within its coverage range, and receiving the tag responses in such micro-zones. Hence, each fielder defines a micro-zone within the coverage range of an RFID reader. Besides, each fielder communicates with the reader through a high-speed channel like one based on IEEE 802.15.4. This brings the advantage of accounting with two independent communication channels. In this approach, there may be from two to 16 fielders in the coverage area of an RFID reader. Previous work shows that as the number of fielders increases, this approach improves the identification process in an RFID network [7].

The second important contribution on this new paradigm for RFID systems is presented in [8]. Here, the authors extend the coverage area of an RFID reader and use several receiving devices. On one hand, the idea is to replace the RFID reader with another device with a larger coverage area. On the other hand, several RFID listeners are used in order to receive the responses sent by tags. An RFID listener is a device designed to operate with two interfaces, one to communicate with RFID tags and the reader, and another one to communicate with subscriber devices. The aim is that an RFID listener can communicate with the tags and the reader through an RF channel as well as with other type of devices through a high-speed channel. The main functions of an RFID listener are to collect tag responses and provide feedback to the reader. In contrast with the approach in [6], there is no limit on the number of RFID listeners in the coverage area of the reader. Even if the work in [8] seems very promising, the authors do not evaluate the performance of their proposal and do not argue about the viability of using such an approach. Thus, in [9], the possibility of using it is evaluated.

In this paper, an analysis is provided to determine which identification mechanism is faster, easy to use, and cheaper to implement on systems with an extended coverage area. In order to do so, in this paper, a detailed evaluation of the existing RFID identification mechanisms for large scale applications is provided. Thus, the MR-Scheme is firstly described, which to date is the only choice for this type of systems. Then, the MR-Scheme is compared with recent alternative proposals. The rest of this paper is organized as follows, in Section 2 the work related with the identification process and the identification mechanisms evaluated in this paper is presented, i.e., the multi-reader scheme and a scheme based on the inclusion of listeners. In Section 3, the parameters and devices considered to model the identification mechanisms analyzed are described. Besides, in the same section, an anti-collision protocol for distributed identification mechanisms based on the EPC Class 1 Generation 2 standard is presented. In Section 4, the parameters needed to evaluate distributed identification mechanisms are presented, such as network deployment and the anti-collision protocols used. Section 5 describes the results obtained in the evaluation. Finally, conclusions and future work are sketched in Section 6.

## 2. Related Work

RFID networks arise under a centralized approach. All the information from the tags goes to the reader; correspondingly, all the information requests come out from the reader. This produces a communication problem known as a bottleneck, which reduces the network performance and has a considerable impact on the processes executed on the network. To solve this issue, most of the existing work focuses on the design of efficient communication protocols. Only few contributions propose to modify the underlaying architecture of RFID networks.

In this work, we focus our study on the identification process and its performance on different RFID network architectures. Among the several processes performed on an RFID network, the identification process is clearly the most important one. It is executed at least once on every RFID system. It consists of collecting all the tag IDs present in an interest area. According to the type and number of the used devices on the RFID network, the identification process may be executed following different reading protocols. In this section, the different ways to execute it according to the different schemes available are described.

### 2.1. Multi-Reader Scheme: MR-Scheme

A large set of RFID applications requires several readers to cover a given area, these applications implement what is called a Multi-Reader (MR) scheme. Such a mechanism is one of the most studied methods to identify a large set of objects in the interest area. An MR-Scheme is composed of a set of readers and a set of tags to be identified. Readers are deployed over an interest area such that they are able to provide a complete coverage. The MR-Scheme comes with some extra interference problems that are not present when using a scheme with one single reader, they may be summarized as:

- Reader-to-reader collisions. This problem, also known as frequency interference, occurs whenever a reader tries to catch a signal from a tag and is masked by the signal from a nearby reader.
- Reader-to-tag collisions. This problem occurs when two or more readers try to simultaneously access a tag. This phenomenon may occur when the tag is in the coverage area of more than one reader, i.e., within an overlapping area.

There is extensive work that solves the MR-Scheme problems. The paper in [10] reviews several contributions; moreover, the following taxonomy is sketched:

- Anti-collision algorithms based on coverage. These types of algorithms aim to minimize the overlapping areas between the readers. To do this, each reader adjusts its transmit power in order to adapt its communication range. A central node is needed to coordinate the whole set of readers.
- Anti-collision algorithms based on resource allocation. They reserve and allocate the resources available to a reader, e.g., frequency, time, bandwidth. Resource allocation is made according to the application requirements or as fair as possible.
- Anti-collision algorithms based on control mechanisms. These algorithms use control packets to indicate that a reader is communicating with a set of tags.

Depending on the application and the network deployment, the use of one of the techniques discussed above may be more adequate than the others. For example, if the readers deployment is known, then algorithms based on coverage may be used; if the reader’s deployment is unknown as well as the readers’ specifications, then algorithms based on resource allocation may be used, but if it is known that the readers account with enough resources, such as more than one antenna, for example, then algorithms based on control mechanisms may be used. Moreover, EPCGlobal under the European regulation, recommends ETSI EN 302 208 [11] as the standard protocol to solve collisions for the MR-Scheme. A distributed mechanism allows readers to operate independently from each other. In EPC-ETSI, each reader chooses, randomly, one of four available frequencies to achieve the communication between a reader and tags. Each reader listens the selected channel for at least 5 ms. If the channel remains free, the reader uses the selected frequency and starts the identification process by sending electromagnetic waves to create a new exploration area to identify RFID tags. Once the reader sends each query packet, it waits for tag responses in a 10 ms interval. If the reader receives no response in that period of time, it frees the channel and it randomly chooses another one. Otherwise, during 4 ms the reader continues the identification process. At the end of such a time interval, the reader frees the channel and waits 100 ms before choosing another channel.

### 2.2. Distributed RFID Sensing

The distributed sensing approach is presented for the first time in [8]. The proposal separates the reception and transmission functions from the reader. It considers the inclusion of a new device into the RFID network: the RFID listener. Additionally, the authors consider replacing the RFID reader by a new device called Illuminator. Thus, an RFID network in the L-Scheme is composed by three types of devices:

- Illuminator. This device is very similar to an RFID reader. An Illuminator does not receive responses from the tags, it just energizes them and sends queries. The Illuminator is envisioned to account with more transmission power than an RFID reader, and therefore, a larger coverage range.
- Listener. The main function of this new device is to collect responses from the tags. In order to do so, firstly the RFID listener needs to decode the Illuminator’s commands. To collect the tags’ responses, an RFID listener may use cooperative reception techniques as is discussed in [8]. These kinds of techniques enable the RFID listener to retrieve useful information from a collision. Thus, the RFID listener acts as an intermediary between the Illuminator and tags since it can decode signals coming from both of them and is able to relay information from the tags to the Illuminator. This last feature makes the RFID listener a very helpful device during the deployment of an RFID network.
- Tags. These are devices with a unique ID, they are attached to the objects to be identified. They are very limited on storage, communication, and processing power.

**Figure 1:**
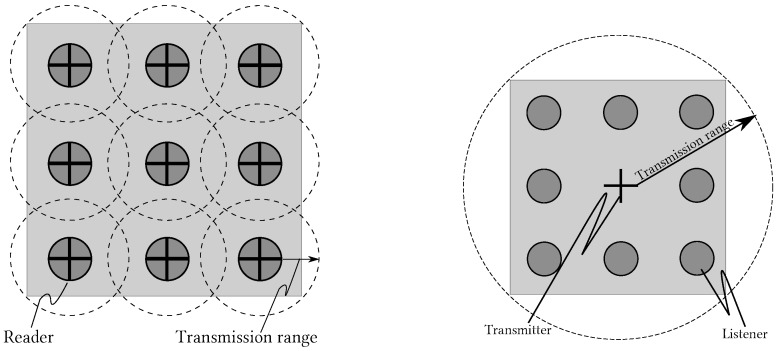
Multi-reader scheme (**left**) and L-Scheme with One Illuminator (**right**) [8].

This new mechanism is called L-Scheme; it increases the coverage range of an RFID system by replacing the traditional RFID reader with an Illuminator. This feature makes the L-Scheme very attractive for applications covering large surfaces with dense scenarios. In the traditional scheme, such applications require more than one reader to cover the interest area; this is known as the MR (Multi-Reader)-Scheme, as mentioned above. Thus, one of the main contributions of the L-Scheme is to reduce the transmission coordination required by the MR-Scheme. Figure 1 compares the deployment of the L-Scheme and the MR-Scheme. As one can see, the main idea is to replace the MR-Scheme with the L-Scheme. This is because in the former there are two extra collision issues that may be avoided by implementing the latter: reader-to-reader and reader-to-tag collisions.

#### Cooperative Reception Techniques

Cooperative reception techniques are able to recover information from a collision. In that sense, there exist several of such techniques that exploit the characteristics of the physical medium to correctly recover a signal. Other techniques are able to recover a signal from a collision if they know one of the colliding signals. We may list such techniques as follows:

- Collision recovery. These mechanisms take advantage of collisions by seeing such phenomenon as an advantage. Surprisingly, RFID tag collisions are destructive and useless [12]. In the literature, there is work focused on multipacket reception for RFID systems [13]; however, most of such contributions suppose important modifications to the RFID devices in the network, and these modifications need to be implemented on the reader [14].
- Interference cancellation. Mechanisms for interference cancellation provide the reader with the ability to suppress the known signals from a colliding one [15], allowing the correct decoding of more than one signal. On one hand, such a function assumes additional features on the reader, e.g., extra memory to store the obtained results from previous queries, while on the other hand it increases the reading rate at an additional cost on processing time [16]. There exist several interference cancellation techniques such as the capture effect [17] that allows identification of an RFID tag even during a collision. Another example is successive interference cancellation [18] that allows identification of an RFID tag by storing a colliding signal and subtracting from it a known one.

Besides, the authors in [8] argue that the L-Scheme may enable dense deployment, reduce cost, and improve the temporary efficiency of RFID systems, however, they do not provide any proof of such affirmation. Also, there is no evidence of the cooperative reception techniques used in that work, which hardens a potential reproduction of the experiment. Additionally, despite of the advantages described by the authors, they only focus on the design and the correct operation of the RFID listener, leaving aside any performance evaluation of the identification scheme proposed therein.

In [9], the authors prove that the coverage range of an RFID system may be extended. The relation between the coverage range of a traditional RFID system and the augmented RFID system is found as being 1:3. Additionally, it is proved that the execution of the identification process can be performed in the L-Scheme with good results.

To our best knowledge, there exist two versions of RFID listeners: a common RFID listener [8] and an augmented RFID listener [2]. The former is the first version of an RFID listener, which is called the S-RFID listener. It covers a reduced area of approximately 3.5 m, which is due to the hardware and software limitations for which the RFID listener was implemented. The latter is an augmented version of an RFID listener, which is called an A-RFID listener. It includes an extra interface to acquire and digitize the RF signal of interest, which helps to increase the maximum reception range up to 12 m. Thus, there are two versions of the L-Scheme, the SL-Scheme that uses S-RFID listeners and the AL-Scheme that uses A-RFID listeners.

### 2.3. Discussion

The inclusion of new devices in the RFID network is the base of a new approach for identification mechanisms in augmented RFID systems. As discussed in [8], these new identification mechanisms require the design of novel anti-collision protocols that exploit all their advantages. Some of these new identification schemes extend the coverage range of the RFID system [8], while the rest propose to preserve its coverage range [6].

In [9], it is validated the possibility of implementing the L-Scheme. In the same work, a reading protocol is proposed and evaluated along with the SL-Scheme in terms of delay. Despite the impact of the results obtained in such a proposal, it is not clear which identification mechanism is better, the SL-Scheme or AL-Scheme. Additionally, the advantages of using the L-Scheme over the MR-Scheme are still unclear. This is because there is not an extensive comparison between these two identification mechanisms. Therefore, a deep study and evaluation of the L-Scheme is highly desirable. Such an evaluation needs to consider the impact of the overlapping areas, the use of cooperative reception techniques, and a dense deployment of listeners. Additionally, it needs to take into account comparisons with different versions of the MR-Scheme: the MR-Scheme with different anti-collision protocols and the MR-Scheme with cooperative reception techniques.

## 3. Towards an Extended Distributed RFID Sensing Protocol

The L-Scheme replaces the identification mechanism based on several readers with one based on one transmitter and several RFID listeners. Such an architecture implies that only one transmitter needs to account with enough resources to cover an interest area. On one hand, this distributes the identification process along the interest area. On the other hand, the bottleneck problem remains for the case of only one transmitter, since it needs feedback from the RFID listeners to perform the identification process in an efficient way.

As mentioned in [8], the maximum coverage range of an RFID system is given by $\min(R_{L},R_{T})$, where $R_{L}$ represents the maximum tag’s reception range and $R_{T}$ represents the maximum energization range. $R_{L}$ refers to the maximum power backscattered by the tags, while $R_{T}$ refers to the maximum power transmitted by the reader. In order to extend the coverage range of an RFID system, the interest is in the cases when $R_{L}\leq R_{T}$. These cases are associated to a particular feature of RFID tags: the tag sensibility. Such a feature may be defined as the minimum energy required by a tag to activate its chip [19]. Then, tag sensibility is an essential parameter to determine the maximum distance from which a tag can be detected.

Additionally, there are two versions of RFID-listeners: S-RFID listener and A-RFID listener. The former has a limited coverage area of around 3.5 m [8], while the latter has an extended coverage area of around 12 m [2].

### 3.1. System Model

In [8], the L-Scheme is evaluated with an ALR-8800 reader along with two ALR-9610 circular antennas and eight 9640 Alien tags [20]. Although the provider offers some information about the capabilities of these devices, such information does not allow to determine the theoretical limit of the system.

Nowadays, we may find a lot of providers of RFID technology. One such provider is Impinj [21]. This company offers a wide variety of RFID products, from RFID readers to RFID tags. Thus, a couple of products is chosen to model the L-Scheme and compute the maximum theoretical coverage range of an RFID system. Here, it is important to remark that a similar study may be done with devices from other providers. The aim of choosing specific devices is just to model the L-Scheme according to existing devices.

In order to model our passive RFID system, the following devices are chosen: a “Speedway revolution” reader [22], “Threshold-FS” Antennas [23], and “Monza 5” tags [24] from Impinj’s Monza family. Table 1 summarizes the important features of the selected devices.

In a previous work, it is found that the coverage range of an RFID system is 15 m, and so in this paper it is fixed to such value. For the L-Scheme, the maximum coverage range is set to 25 m, which is the maximum supported value by current commercial RFID technology.

### 3.2. Anti-Collision Protocols for Distributed Sensing

With the advent of new RFID identification mechanisms, a great need for new anti-collision protocols arises [8]. Even if the most important contribution of such new identification mechanisms proposes to separate the transmission and reception functions from a reader, all the anti-collision protocols need feedback from the network to improve the resource usage. In [9], a modified version of the EPC Class1 Generation2 standard [25] for the L-Scheme is proposed, which is renamed in this paper as EPCGen2-LS.

#### 3.2.1. The EPC Class 1 Generation 2 Protocol

The EPC Class1 Generation2 or EPC Gen2 is the standard used in passive RFID environments. It is based on an ALOHA protocol known as DFSA (Dynamic Frame Slotted ALOHA) [26], which considers that time is slotted and grouped into frames. EPCGen2 works as follows: the reader begins the identification process by sending a *Query* packet containing the size of the current frame, $f=2^{Q}-1$, with Q∈ [0, 15]. At the reception of such packet, tags choose a time slot according to a uniform distribution, k∼U(0,f). Tags choosing k=0 send an *RN16* packet to the reader, which produces any of the following three cases:

- No response. There is no response to the current query, which means that no tag selected the current slot in the frame.
- Unique response. Only one tag selects the current slot, allowing a correct decoding of the *RN16* packet in the reader. When this happens, the reader sends an *ACK* packet to indicate to the corresponding tag that it has been identified. In turn, the tag responds to the reader with its ID, which completes the identification of such tag.
- Collision. If more than one tag responds in the current slot, a collision will be produced. In this case, the reader is not able to decode the *RN16* packet sent by the tags.

Following any of the cases just described, the reader continues the identification process with the transmission of a QueryRep packet. The process just described continues slot by slot. At the end of the frame, the reader counts the number of empty, single, and collision slots in order to estimate the number of unidentified tags. Next, the reader executes a mechanism to adapt the length of the subsequent frame. Then, the reader transmits a QueryAdj packet with a new frame-length to continue the identification process. Thus, the reader continues the process until no more responses to the queries are received. It is important to note that EPCGen2 uses a different frame-length in each identification cycle, which improves the identification process.

#### 3.2.2. The EPCGen2-LS Protocol

EPCGen2-LS is standard compliant, which means that it preserves the commands recommended by the standard. In the following sections, the operation of the EPCGen2-LS protocol is described in detail.

Table 2 summarizes the commands used by EPCGen2-LS. The commands for the transmitter are the same as those an RFID reader typically uses during the identification process. There are two commands for RFID listeners: one to indicate a collision event or a no response event, and the other one to signal an identification. It is possible to avoid the use of a command to indicate a no response event, however this would be very complicated. On one hand, there should be a high coordination between the participant devices. On the other hand, the use of a sophisticated method to determine a no response event would be needed. Finally, the tags commands in the L-Scheme are the same as those recommended by the standard.

Since EPCGen2-LS is a DFSA-based protocol, it requires a mechanism to adapt the frame-length during each identification cycle. It means that an estimation function is needed to resize the frame length according to the estimated number of unidentified tags [27].

Unlike other identification mechanisms [6], the L-Scheme employs techniques to retrieve useful information from a collision. This causes a problem when the Illuminator tries to determine the next frame-length since a query does not generate the same result in each RFID listener.

In EPCGen2-LS, a time slot is considered as empty when there is no response in all the RFID listeners. In the same way, a time slot is considered as successful when there is at least one RFID listener with an identification. Finally, a collision time slot is identified when there is more than one response in each of the RFID listeners, even if there have been identifications of such collisions. Since the RFID listeners are responsible for collecting the tags’ responses, they need to inform the Illuminator of the result of each query. An RFID listener may send one Reply command or two RN16 commands by query. In EPCGen2-LS, the Illuminator manages independent counters for each RFID listener. On one hand, this improves the protocol performance. On the other hand, this requires the storage of a great amount of information when the number of RFID listeners is large.

Thus, the mechanism to adapt the frame-length for EPCGen2-LS is based on the estimation function proposed in [28], it uses information from all the RFID listeners. This means that according to the number of collision slots reported in an RFID listener, the Illuminator computes the corresponding value of *Q* according to the estimated number of unidentified tags on each RFID listener. The Illuminator selects the highest value among the set of values calculated for each RFID listener and sets the frame-length for the next identification cycle equal to such a value.

The operation of the EPCGen2-LS is presented in Algorithm 1. The pseudocode presented serves as a guide for the implementation of the EPC Gen 2-LS protocol. Since it is standard compliant, it does not require any modification on the RFID tags, it only requires programming the Illuminator and the RFID listeners, which are reprogrammable devices [8,10]. The pseudocode comprises the procedures executed by the devices that participate in the L-Scheme:

Illuminator’s procedure. It comprises from line 1 to 28 of Algorithm 1. The Illuminator defines the initial frame-length in lines 1–2. Then, it starts the identification process in line 3 and initializes the independent variables for each RFID listener, in lines 5–9. Thus, the Illuminator collects the result of the current slot in each RFID listener. In case of collision or no response, in lines 15–18, the Illuminator increases its corresponding counter. In case of an identification, the Illuminator sends an ACK packet to the corresponding tag and waits for the tag’s ID in lines 20–22. Once the Illuminator finishes the process of collecting results, it begins a new slot sending a QueryRep packet in line 23. The process just described continues slot by slot until the end of the frame. When the current identification cycle finishes, the Illuminator estimates the number of unidentified tags on each RFID listener and then determines their associated *Q* value, in lines 23–26. Now, the Illuminator computes the frame-length for the next identification cycle determining the value of *Q* in line 27. Finally, the Illuminator continues the process by sending a QueryAdj packet with a new *Q* value, in line 28. This process continues until all the tags have been identified.

RFID listener procedure. An RFID listener waits for Illuminators commands, in line 30. If the command is a query, the RFID listener waits for tag responses in line 32. In a collision event, in lines 34–35, the RFID listener sends a Reply packet to the Illuminator and verifies if it can recover the collision in line 36. In case of a collision recovery, the RFID listener sends the RN16 command to the Illuminator in line 38, and then waits for the corresponding ACK in line 39. When this occurs, the RFID listener waits the EPC-ID packet from the tag, in line 42. In line 43, the RFID listener retransmits to the Illuminator the received EPC-ID from a tag. In case of only one response, in lines 44–48, the RFID listener sends the RN16 command to the Illuminator and waits for an ACK packet from the Illuminator. After receiving such packet, the RFID listener waits for the EPC-ID command from the tag and retransmits it to the Illuminator. In case of no response, the RFID listener sends a Reply packet to the Illuminator, in line 50.

Tag’s procedure. The tag waits for Illuminator commands in line 53. Once a tag receives a command, it verifies if it is a query beginning a new identification cycle, i.e., a Query or a QueryAdj command, in line 54. In such a case, the tag computes the next frame-length, *f*, in line 55. The tag selects a slot in the interval [0, *f*] following a uniform distribution and loads such value into its counter *k*, in line 56. In case of a QueryRep command, the tag decrements its counter *k* in line 58. In case of an ACK, the tag send its ID and changes its state to Detected in lines 60 and 61, respectively. Finally, in each received message the tag verifies, if its counter reaches k=0, it sends its the corresponding RN16 in lines 62–63.
**Algorithm 1** Pseudo-code of the EPC Gen2 protocol for large scale RFID systems [9].**Illuminator procedure**
1:$Q_{ini}=4.0$← Initial *Q* value2:$Q=Q_{ini}$
3:Send(Query,Q)
4:**while** (∃ *Tag to be identified*) **do**
5: $E_{tags}\left[k\right]=0$← Estimated number of tags in listener *k*6: Q[k]=0← *Q* value for the *k*-th listener7: N_Empty[k]=0← Number of empty slots for listener *k*8: N_Single[k]=0← Number of single slots for listener *k*9: N_Collision[k]=0← Number of collision slots for listener *k*10: $L=2^{Q}$← Frame length11: **for**
i=1 to L **do**
12:  **for**
***all** RFID listeners*
**do**
13:   Receive(Pckt)
14:   **if** (Pckt==Reply) **then**
15:    **if** (Reply==Collision) **then**
16:     N_Collision[k]=N_Collision[k]+1
17:    **else**
18:     N_Empty[k]=N_Empty[k]+1
19:   **else**
20:    N_Single[k]=N_Single[k]+1
21:    SEND(ACK)
22:    WAIT_ID
23:  Send(QueryRep)
24: **for**
***all** RFID listeners*
**do**
25:  $E_{tags}\left[k\right]=2.39*N\_Collision\left[k\right]$← Estimation function [28]26:  $Q\left[k\right]=FINDFRAME(E_{tags}\left[k\right])$← Select the optimal value of *Q* [29]27: Q=max(Q[k])
28: Send(QueryAdj,Q)
**Listener procedure**
29:**while** (Commands) **do**
30: Receive(Cmd)
31: **if**
Cmd==(Query|QueryRep|QueryAdj)
**then**
32:  $Wait(Query_{Timeout})$
33:  **if** (Response) **then**
34:   **if** (Collision) **then**
35:    Send(Reply)← Collision event36:    **if** (**Can be recovered?**) **then**
37:     **for**
***all***
**identifications do**← Could be one or two identifications38:      Send(RN16)← The RN16 is reported to the transmitter39:      Receive(Cmd)
40:      **if** (Cmd==ACK) **then**
41:       WAIT_ID← Wait for an ID from a tag42:       Send(ID−EPC)← The ID is reported to the transmitter43:   **else**← Identification case44:    Send(RN16)
45:    Receive(Cmd)
46:    **if** (Cmd==ACK) **then**
47:     WAIT_ID
48:     Send(ID−EPC)
49:  **else**
50:   Send(Reply)← No response case**Tag procedure**
51:*Detected* = *false*
52:**while not Detected do**
53: *Receive*(*Message*);
54: **if** (*Message* == *Query*|*QueryAdj*) **then**
55:  *f* = 2^*Q*^ − 1
56:  *k* = *U*(0, *f*)
57: **else if** (*Message* == *QueryRep*) **then**
58:  *k* = *k* − 1
59: **else if** (*Message* = *ACK*) **then**
60:  *Send*(*ID*)
61:  *Detected* = *true*
62: **if** (*k* == 0) **then**
63:  *Send*(*RN*16);


## 4. Evaluation

In order to compare the two existing approaches for large-scale RFID systems, scenarios are configured for a fair evaluation, taking into account several parameters that highlight their advantages and limitations. To do so, first the size of the interest area is determined based on the maximum coverage area of an Illuminator, with the features described in Table 1. In this way, the evaluation is focused on applications requiring to cover a large interest area, such as a warehouse, departmental store, or points of arrival of packages, just to mention a few. Then, a model that allows to determine the total number of receiving devices being required in each approach to completely cover such an area is proposed. Finally, the two approaches are compared in terms of identification delay and reading throughput. The first measure refers to the time an RFID scheme spends in identifying the whole set of tags in the interest area. The second measure is the number of tags read per second. Since the use of cooperative reception techniques is considered on the evaluated identification schemes, an evaluation of the number of collision slots and the total number of used slots is done to understand the advantages of implementing such techniques. Besides, the evaluation assumes that there is no source of interference since this type of phenomenon has an impact on every identification mechanism by adding time to the identification process. Additionally, since a numerical evaluation is performed, it would not capture the whole essence of such phenomenon.

In this way, the evaluation considers different parameters for a better understanding of the efficiency of an identification scheme, either from a practical point of view or from a theoretical perspective. Therefore, the L-Scheme is compared in its two versions with the MR-Scheme considering the aspects mentioned above.

### 4.1. System Model

The propagation models for RF signals are themselves a research topic. Even if they are part of the RFID systems modeling, they are not a central issue in this research. One of the basis for modeling the propagation signals in RFID systems is the spherical approach [3,4,5]. Taking into account such an approach along with the devices described in Table 1, an interest area (IA) of 1963.5 $m^{2}$ is considered. To cover such area, depending on the employed identification mechanism, one might need more or less devices to guarantee a total coverage. 

#### Network Deployment

**Figure 2:**
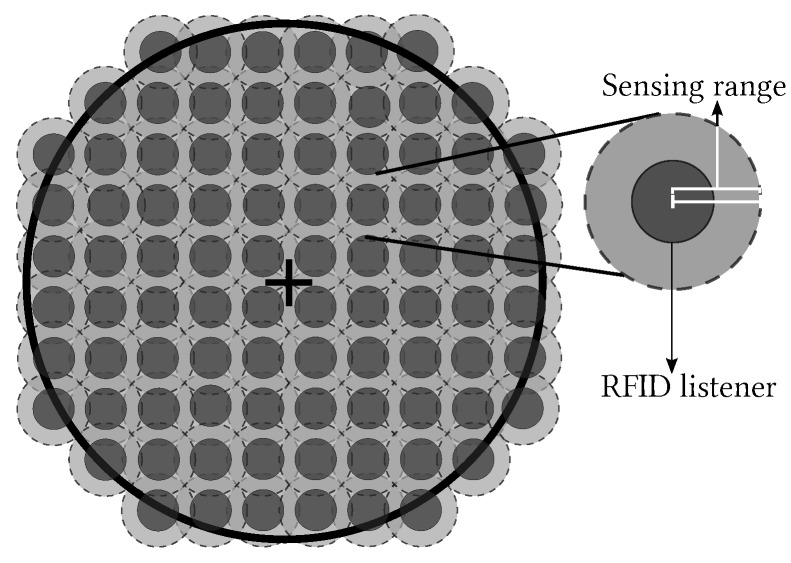
Deployment for the SL-Scheme.

**Figure 3:**
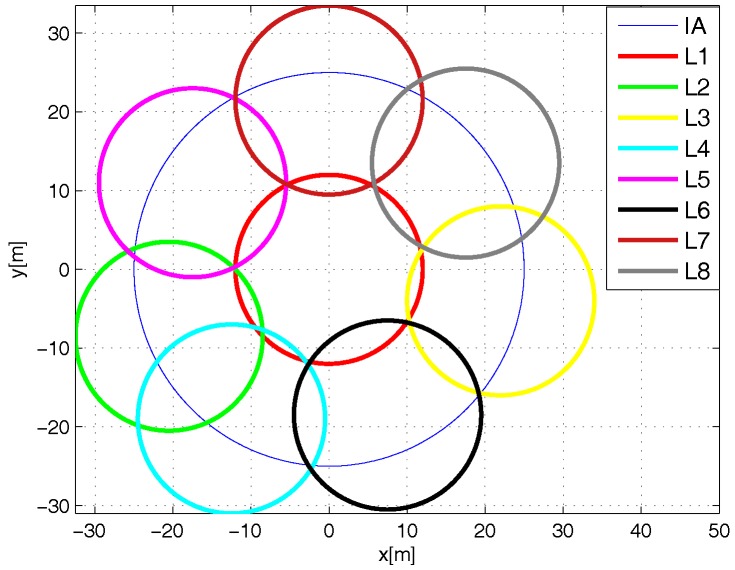
Configuration approximated with the genetic algorithm for the AL-Scheme.

**Figure 4:**
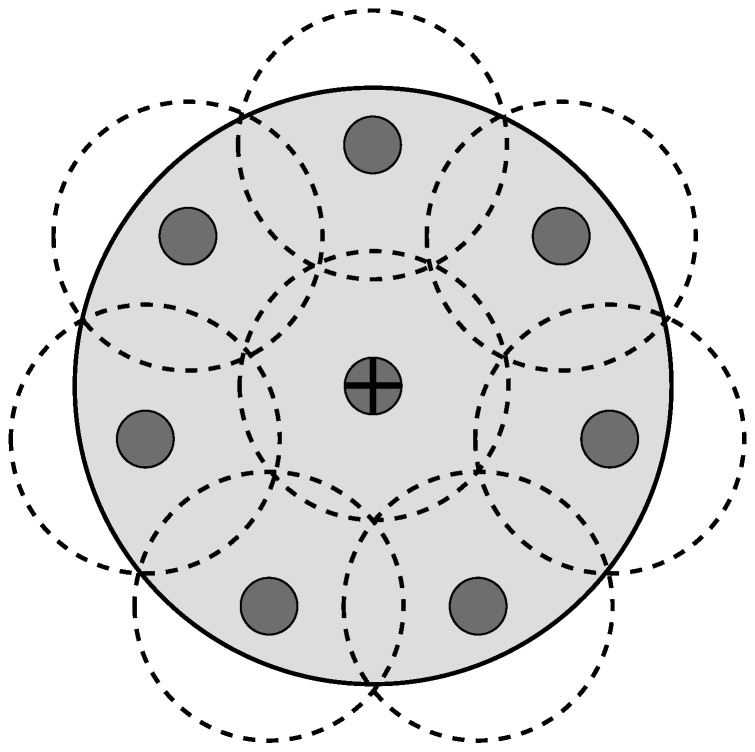
Optimum configuration deployment for eight listeners.

There are two versions of the L-Scheme: the SL-Scheme and AL-Scheme. In Figure 2 and Figure 3 the deployment of the SL-Scheme and AL-Scheme is presented, respectively. Notice that the SL-Scheme requires deployment of over 80 RFID listeners to guarantee a total coverage, while the AL-Scheme requires eight RFID listeners to guarantee the same. For convenience, the configuration deployment for the SL-Scheme is selected in that way, while for the AL-Scheme it is obtained from a genetic algorithm [30]. Figure 4 shows the optimum configuration deployment reported in the literature when the ratio $0.437\leq R_{I}/R_{L}\leq 0.5$, where $R_{I}$ is the radius of the Illuminator’s coverage range and $R_{L}$ is the radius of the sensing range of an RFID listener. Figure 4 details the configuration deployment approximated with our genetic algorithm.

**Figure 5:**
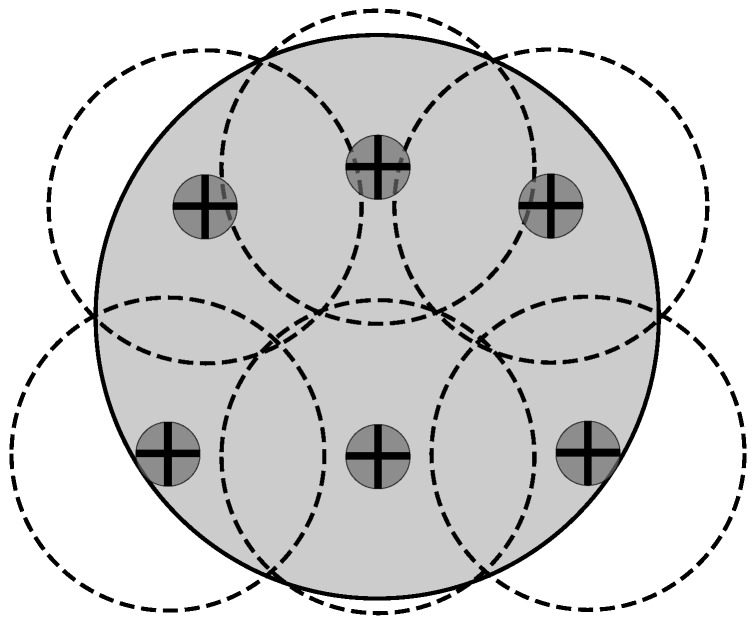
Optimum configuration deployment for six readers.

**Figure 6:**
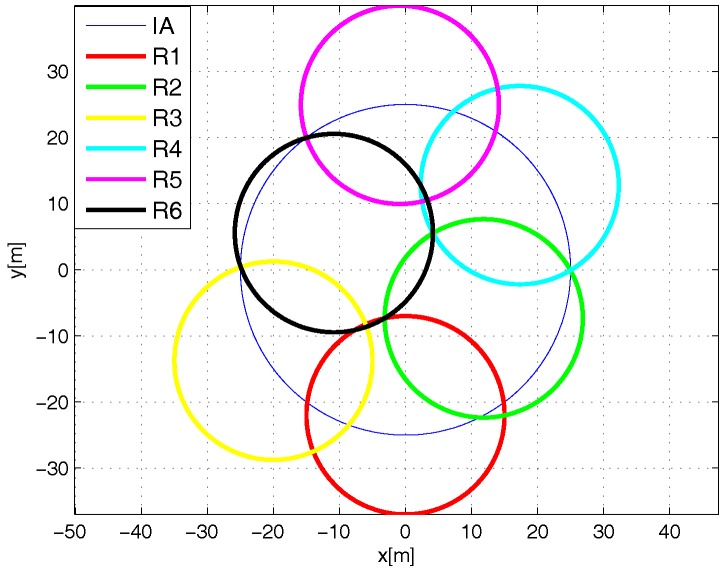
Configuration approximated by genetic algorithm for the Multi-Reader scheme.

The configuration deployment for the Multi-Reader Scheme is presented in Figure 5 and Figure 6. As one can see, only seven readers are needed to completely cover the interest area. Again, the configuration deployment is obtained with our genetic algorithm. Figure 5 shows the optimum configuration reported in the literature with a deployment of six devices according to the ratio $0.55\leq R_{I}/R_{R}\leq 0.61$ [31], where $R_{R}$ is the radius of the coverage range of an RFID reader. Figure 6 shows the configuration deployment of the Multi-Reader Scheme approximated with our genetic algorithm.

The number of tags in the interest area varies from 1000 to 12,000 tags. The number of tags is determined considering 2000 tags per RFID reader, which is a common configuration for passive RFID systems [32,33]. The tags distribution is application dependent, however, it is possible to find three general cases about this issue:

- Best. For the L-Scheme, the number of tags in the sensing range of an RFID listener is the same for each of these devices. Regarding the Multi-Reader scheme, the best case occurs when the number of tags in the coverage range of an RFID reader is the same for each RFID reader. Additionally, there are no tags in the overlapping areas. This case eliminates the reader-to-tag collision events that occur with the MR-Scheme, which is not very representative of an RFID application.
- Worst. The whole set of tags is in the sensing range of an RFID listener for the L-Scheme or it is in the coverage range of only one RFID reader for the MR-Scheme. On one hand, this case vanishes the multi-reader concept. On the other hand, this case does not make any sense for a comparison between distributed identification schemes.
- Average. For the L-Scheme, the number of tags in the sensing range of an RFID listener is proportional to its coverage area. For the MR-Scheme, the number of tags in the coverage area of an RFID reader is proportional to its coverage area. In this case, there are overlapping areas and tags in such areas. On one hand, this produces reader-to-reader collisions and reader-to-tag collisions on the MR-Scheme. On the other hand, this allows the exploitation of cooperative reception techniques from the L-Scheme. Thus, this is the general case regarding the number of tags per coverage area and, therefore, the most interesting case to evaluate.

Based on the cases described above, the tags are distributed within the interest area following a uniform distribution. This guarantees an equitable comparison concerning the number of tags per RFID listener and per RFID reader.

### 4.2. Anti-Collision Protocols and Simulation Parameters

The EPCGen2-LS protocol is used for both the L-Scheme and the AL-Scheme. For the MR-Scheme, a sequential anti-collision algorithm is used to avoid reader-to-reader and reader-to-tag collisions. This is because we do not assume special features from the readers. Besides, in order to implement anti-collision algorithms for the MR-Scheme, it is necessary to separate readers about tens to hundreds of meters away from each other in order to guarantee no interference [34]. Finally, for the MR-Scheme, the EPCGen2 standard is used to solve collisions between tags.

For the proposed EPCGen2-LS protocol and the EPCGen2 standard, the estimation function presented in [28] is employed and Table 1 from [29] as mechanisms to adapt the frame-length during each cycle. Additionally, the delay of each protocol is approximated assuming that the number of subcarrier cycles per symbol is M=2.

Since the authors of the L-Scheme do not consider a particular cooperative reception technique, one for collision recovery and another one for interference cancellation are chosen. The collision recovery technique in [13] is employed, which allows recovery with a probability of 85% the signals from two colliding tags. Regarding the interference cancellation technique, an approach based on the removal of known signals is followed, i.e., when an RFID listener decodes correctly an *RN16* packet, it broadcasts it to its neighbors so that they can remove such a signal from the received one. Also, we consider that RFID listeners are integrated to wireless nodes that are able to communicate between them and with the Illuminator through the ZigBee protocol [35]. Zigbee is based on the IEEE 802.15.4 standard and provides a channel capacity of up to 250 kb/s.

Finally, in order to fairly evaluate the considered mechanisms, three versions of the MR-Scheme are taken into account. The first one considers a sequential anti-collision protocol for readers and the EPC Gen2 for tags, i.e., MR-Seq-EPCGen2. The second one takes into account the features of the MR-Seq-EPCGen2 protocol and adds the collision recovery feature, i.e., MR-Seq-CR-EPCGen2. Finally, the third one considers a “parallel” anti-collision mechanism for readers along with the collision recovery feature and the EPCGen2 for tags, i.e., the MR-Par-CR-EPCGen2. In the last case, the parallelism takes place only for the readers without overlapping areas in common. Therefore, according to Figure 6, R1 and R5 can identify a tag at the same time.

Thus, each identification mechanism is implemented with its corresponding anti-collision protocol in a numerical simulation. MatLab is used to implement the simulation scenario. 500,000 simulations are executed for each set of tags.

## 5. Numerical Results

### 5.1. MR-Scheme vs. L-Scheme and AL-Scheme

**Figure 7:**
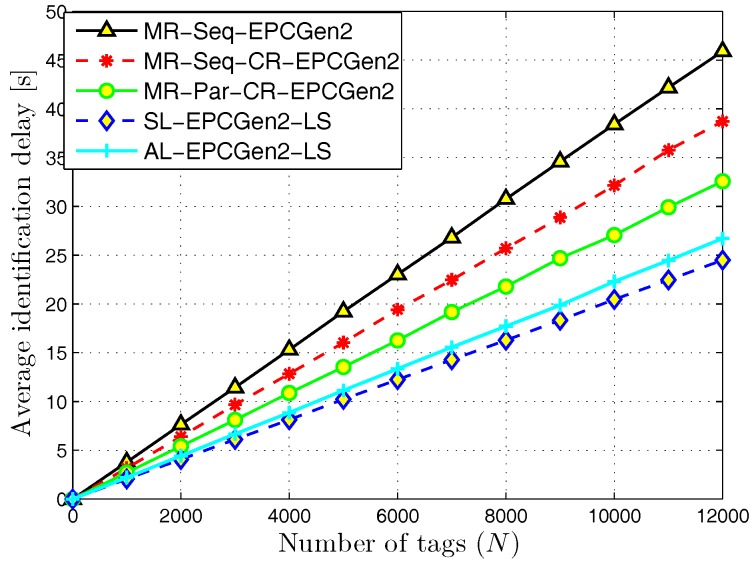
Average identification delay for distributed identification mechanisms.

Figure 7 presents the average identification delay for the MR-Seq-EPCGen2, MR-Seq-CR-EPCGen2, MR-Par-CR-EPCGen2, SL-EPCGen2-LS, and AL-EPCGen2-LS protocols. As one can observe, SL-EPCGen2-LS has the lowest identification delay among all the evaluated identification mechanisms. This is because the probability of identifying one tag with this scheme is higher than with the rest of them. Notice that this result suggests that a dense deployment of RFID listeners is better than a sparse one. However, in practical terms, the SL-EPCGen2-LS protocol is not the best choice if one considers that the reduction obtained in identification delay is only about two seconds compared to AL-EPCGen2-LS. However, the difference between the number of used devices with SL-EPCGen2-LS compared to AL-EPCGen2-LS is about 80.

**Figure 8:**
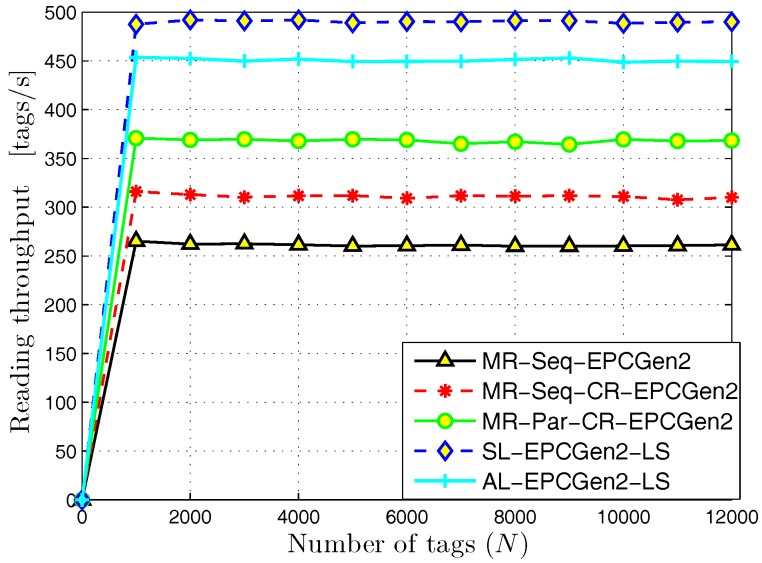
Reading throughput for distributed identification mechanisms.

The results for the reading throughput are shown in Figure 8. As we can appreciate, the SL-EPCGen2-LS protocol exhibits the highest value, which is close to 500 tags/s. This is due to the high number of RFID listeners deployed in the interest area and the use of cooperative reception techniques. Both facts increase the probability of an identification for each slot in a query frame. On the other hand, observe that the worst performance is obtained with the MR-Seq-EPCGen2 protocol. The reason for this is because it executes the identification process in a progressive way, i.e., it performs the identification process in a given part of the interest area and once it finishes, it continues in a different part.

**Figure 9:**
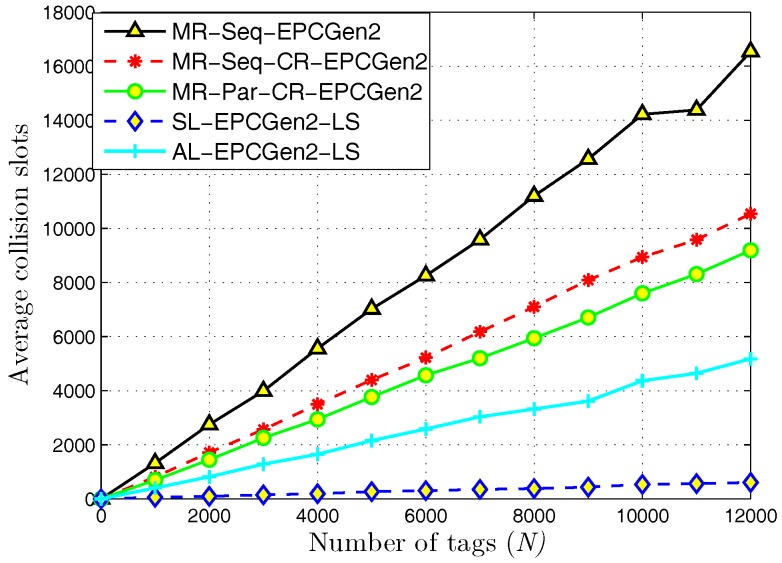
Average collision slots for distributed identification mechanisms.

Figure 9 presents the results regarding average collision slots. Notice that the highest value is obtained with the MR-Seq-EPCGen2 protocol, and the lowest value with SL-EPCGen2-LS. Regarding MR-Seq-EPCGen2, collisions represent a serious problem since this mechanism does not implement a procedure to recover from collisions or to obtain useful information from them. On the contrary, the SL-EPCGen2-LS protocol can take advantage from collision events either by recovering them or by using them to retrieve useful information. From here, one can explain the high reading throughput obtained with SL-EPCGen2-LS, since even if the number of collision slots is low, this protocol transforms most of them in successful slots.

**Figure 10:**
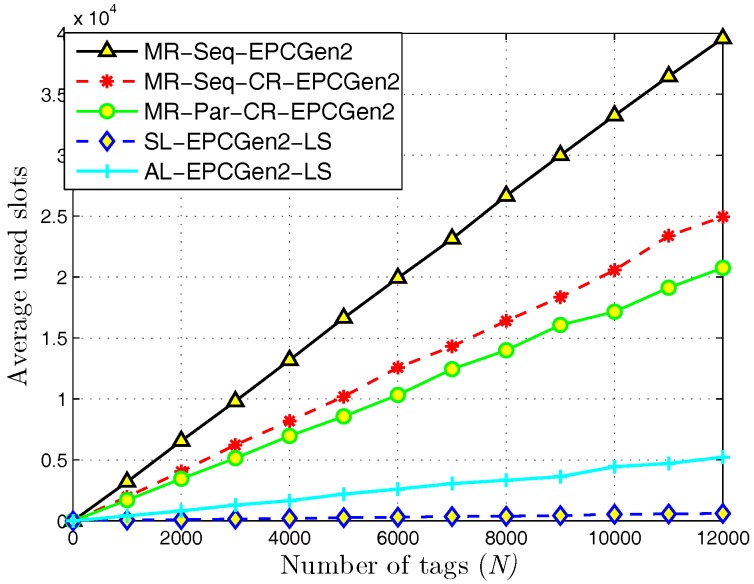
Average used slots for distributed identification mechanisms.

In Figure 10, we show the results for the average used slots for each evaluated identification mechanism. Notice that to perform the identification process in each set of tags, the L-Scheme consumes less slots than the MR-Scheme. For the MR-Scheme, observe that MR-Seq-EPCGen2 has the highest number of used slots. In second place, we find MR-Seq-CR-EPCGen2 which implements the CR technique. In this case, notice that the use of this technique can reduce considerably the number of used slots for an identification mechanism. In third place, we find MR-Par-CR-EPCGen2 which executes the identification process in a parallel way and uses the CR technique. For this case, notice that the number of used slots for MR-Par-CR-EPCGen2 is not very different to the one obtained with MR-Seq-CR-EPCGen2. This suggests that the contribution on time reduction with the CR technique is greater than with the identification process, i.e., there is no big difference on the number of slots by executing the identification process either sequentially or in a parallel way. Regarding the L-Scheme, notice that SL-EPCGen2-LS consumes less slots than AL-EPCGen2-LS. Considering that SL-EPCGen2-LS also has less collision slots than AL-EPCGen2-LS, this result is expected. Moreover, if one considers that the opportunity to identify a tag depends directly on the number of receiving devices deployed along the interest area, it is evident that SL-EPCGen2-LS always has the best opportunities.

### 5.2. L-Scheme and AL-Scheme

**Figure 11:**
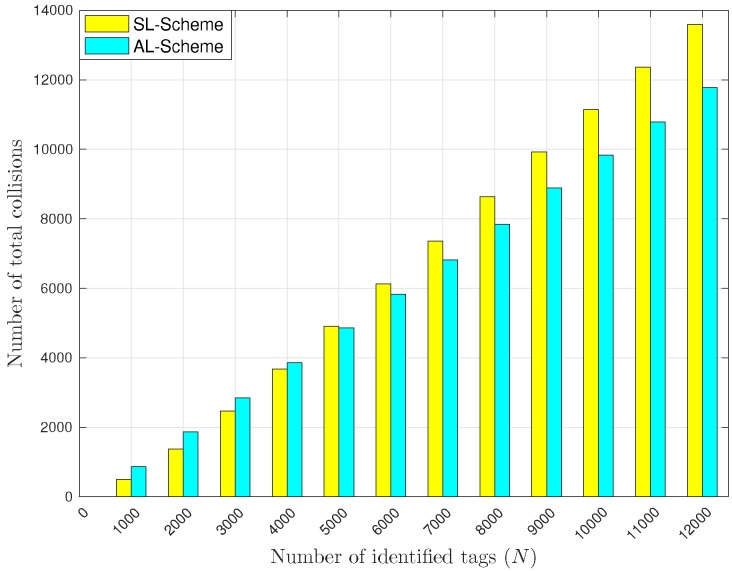
Total number of collisions reported by RFID listeners.

In this section, the two versions of the L-Scheme are compared. Figure 11 shows the total number of collisions reported by all the RFID listeners. Notice that when the number of tags is greater than 5000, the number of collisions with the AL-Scheme is lower than with the SL-Scheme, however, this changes when the number of tags is lower than 4000. Notice that as the total number of tags in the interest area increases, the probability of collision increases as well. Additionally, one needs to keep in mind that the AL-Scheme has less devices deployed for reception in the interest area than the SL-Scheme, which reduces the number of reported events. Thus, it is evident that the SL-Scheme reports more collisions than the AL-Scheme for almost all the evaluated sets of tags, because the former includes more receiving devices to retrieve information from. Hence, when a lot of collisions occur because of a reduced frame size and a there is a high number of tags, the SL-Scheme will incur in a lot of collisions compared to the AL-Scheme.

**Figure 12:**
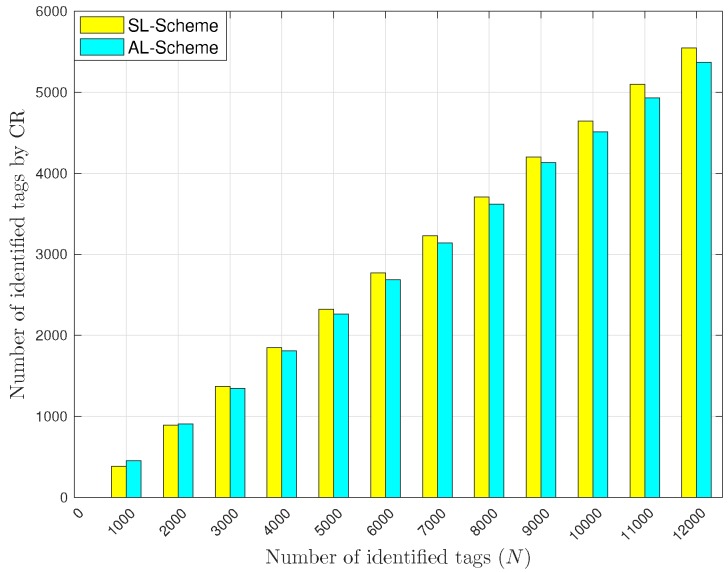
Average number of tags identified with CR.

**Figure 13:**
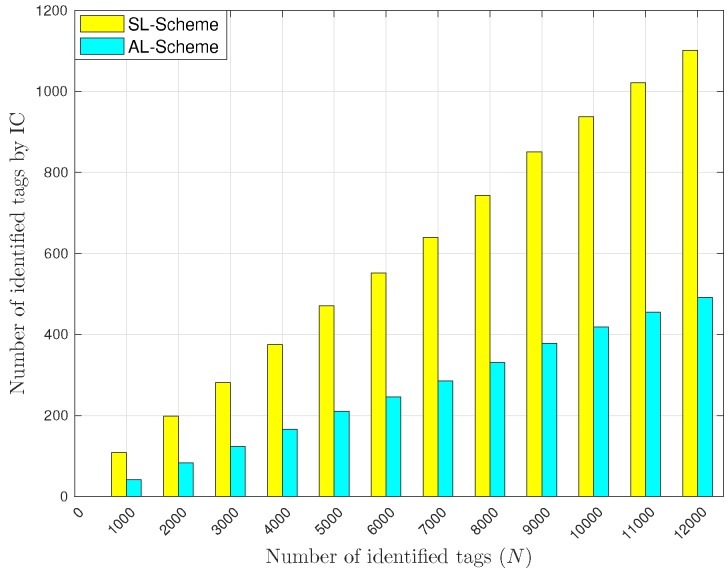
Average number of tags identified with IC.

Figure 12 and Figure 13 show the total number of identified tags with cooperative reception techniques for the AL-Scheme and the SL-Scheme. As we can see in Figure 12, the total number of identified tags with CR is greater for the SL-Scheme than with the AL-Scheme. The cause is that with the CR technique, a collision could occur within in two identifications. Thus, if a lot of collisions occur, the probability of turn them into identifications is high. Also, observe in Figure 12 that the AL-Scheme outperforms the SL-Scheme when the number of tags is lower than 3000. Again, this occurs because in such cases the AL-Scheme reports a greater number of collisions than the SL-Scheme.

Finally, Figure 13 shows the total number of identified tags with the IC technique. As one can see, the number of identified tags is lower than with the CR technique. Also, notice that the SL-Scheme has more identifications than the AL-Scheme. Again, the reason is that the SL-Scheme reports more collisions than the AL-Scheme. In this case, unlike previous cases, observe that the SL-Scheme outperforms the AL-Scheme for all the evaluated sets of tags. Additionally, as the number of tags in the interest area increases, the number of identified tags with the IC technique increases as well. This suggests that the IC technique may be advisable when there is a dense deployment of receiving devices and a lot of collisions.

## 6. Conclusions

The identification process is the most important one in an RFID system. To date, there are several ways to perform this process according to the employed identification method. The centralized identification mechanism seems very adequate for applications with low requirements. However, current applications demand a higher performance and scalability. Distributed identification mechanisms arise as a response to such demand.

In this paper, the L-Scheme has been evaluated, which is a distributed identification mechanism. It has also been compared with the well known multi-reader (MR) scheme. The evaluation was done under identical conditions and as fair as possible according to the current technology. The comparison comprised practical parameters to evaluate any RFID system. The focus was on the time spent by the system to execute a process. The results obtained in this work have shown that the distributed scheme can outperform the multi-reader scheme in identification delay. Additionally, it has been found that when there are more receiving devices in the interest area the identification process can be speed up. This is mainly due to the use of cooperative reception (CR) techniques, which showed a higher performance in dense scenarios with a high number of collisions.

Based on the evaluation performed here, we can conclude that the MR-Scheme can be implemented in distributed scenarios with a reduced set of tags, while the L-Scheme can be implemented in dense scenarios with many tags distributed along an interest area of considerable size.

Finally, about the implementation costs, we need to say that the SL-Scheme imposes an increase on them regarding the MR-Scheme, while the AL-Scheme supposes a reduction on the implementation costs. In that sense, the adequate use of an identification mechanism over another is still unclear. Therefore, we will focus our future work on determining the correct use of an identification mechanism based on the requirements of a set of applications. 

## Figures and Tables

**Table 1:** Impinj devices specifications.

Parameter	Reader	Tag
Model	Speedway revolution	Monza 5
Sensitivity	−82 dBm	−17.8 dBm
Gain	5 dBi	2 dBi
Transmission power	1–3 W	–

**Table 2:** EPCGen2-LS commands.

Command	Code	Length	Description
**Illuminator**			
Query	1000	22 bits	Command sent at the beginning of the identification process
QueryRep	00	4 bits	Command sent at the beginning of a slot
QueryAdjust	1001	9 bits	Command sent at the beginning of an identification cycle
ACK	01	18 bits	Command sent by the illuminator after a successful identification
**Tag**			
RN16	–	16 bits	Command sent by the tag as a response to a *Query*, *QueryRep* or *QueryAdjust* command.
ID-EPC	–	21–528 bits	Command sent by a tag as a response to an *ACK* command.
***RFID listener***			
RN16	–	16 bits	Retransmission of the *RN16* command.
ID-EPC	–	21–258 bits	Retransmission of the *ID-EPC* command.
Reply	–	4 bits	Command sent to indicate a collision or no response event.

## References

[1] Das R. RFID Forecasts, Players and Opportunities 2017–2027. https://www.idtechex.com/research/reports/rfid-forecasts-players-and-opportunities-2017-2027-000546.asp.

[2] De Donno D., Ricciato F., Catarinucci L., Tarricone L. Design and applications of a software-defined listener for UHF RFID systems. Proceedings of the IEEE International Microwave Symposium Digest (MTT).

[3] Ali K., Hassanein H., Taha A.E.M. RFID Anti-collision Protocol for Dense Passive Tag Environments. Proceedings of the IEEE Local Computer Networks (LCN).

[4] Alsalih W., Ali K., Hassanein H. Optimal distance-based clustering for tag anti-collision in RFID systems. Proceedings of the IEEE Local Computer Networks (LCN).

[5] Tseng D.F., Lin Z.C. An Anti-Collision Algorithm in RFID Systems Based on Interference Cancellation and Tag Set Partitioning. Proceedings of the IEEE Vehicular Technology Conference (VTC-Spring).

[6] Ali K., Hassanein H. Distributed receiving in RFID systems. Proceedings of the 34th IEEE Conference on Local Computer Networks (LCN).

[7] Sanchez L., Ramos V. (2017). Efficient distributed identification for RFID systems. Wirel. Personal Commun..

[8] De-Donno D., Ricciato F., Catarinucci L., Coluccia A., Tarricone L. Challenge: Towards distributed RFID sensing with software-defined radio. Proceedings of the 16th ACM Annual International Conference on Mobile Computing and Networking (MobiCom).

[9] Sanchez L., Ramos V. An EPC Class-1 Generation-2 anti-collision protocol for RFID tag identification in augmented systems. Proceedings of the International EURASIP Workshop on RFID Technology (EURFID).

[10] Li Z., He C., Tan H.Z. Survey of the advances in reader anti-collision algorithms for RFID systems. Proceedings of the 50th IEEE Conference on Control and Decision (CCDC).

[11] EPCGlobal (2011). EN 302 208-1: Electromagnetic Compatibility and Radio Spectrum Matters.

[12] Castiglione P., Ricciato F., Popovski P. Pseudo-random ALOHA for inter-frame soft combining in RFID systems. Proceedings of the 18th International Conference on Digital Signal Processing (DSP).

[13] Fyhn K., Jacobsen R.M., Popovski P., Scaglione A., Larsen T. (2011). Multipacket Reception of Passive UHF RFID Tags: A Communication Theoretic Approach. IEEE Trans. Signal Process..

[14] De Donno D., Tarricone L., Catarinucci L., Lakafosis V., Tentzeris M.M. (2012). Performance enhancement of the RFID EPC Gen2 protocol by exploiting collision recovery. Prog. Electromagn. Res. B.

[15] Ricciato F., Castiglione P. (2013). Pseudo-Random ALOHA for Enhanced Collision-Recovery in RFID. IEEE Commun. Lett..

[16] Kumar R., La Porta T.F., Maselli G., Petrioli C. Interference Cancellation-based RFID Tags Identification. Proceedings of the 14th ACM International Conference on Modeling, Analysis and Simulation of Wireless and Mobile Systems (MSWiM).

[17] Kim J.H., Lee J.K. (1999). Capture effects of wireless CSMA/CA protocols in Rayleigh and shadow fading channels. IEEE Trans. Veh. Technol..

[18] Halperin D., Anderson T., Wetherall D. Taking the Sting out of Carrier Sense: Interference Cancellation for Wireless LANs. Proceedings of the 14th ACM International Conference on Mobile Computing and Networking (MobiCom).

[19] Nikitin P.V., Rao K.V.S. Performance limitations of passive UHF RFID Systems. Proceedings of the International Symposium on Antennas & Propagation (ISAP).

[20] Alien Technology Inc.. http://www.alientechnology.com.

[21] Impinj Inc.. http://www.impinj.com.

[22] Impinj Speedway UHF RFID Reader. https://www.impinj.com/platform/connectivity/speedway-r420/.

[23] Threshold FS Antenna Datasheet. https://support.impinj.com/hc/en-us/articles/202755648-Brickyard-Antenna-Datasheet.

[24] Monza 5 UHF RFID TAG CHIPS. https://support.impinj.com/hc/en-us/articles/202756948-Monza-5-Tag-Chip-Datasheet.

[25] EPCGlobal (2008). EPC Radio-Frequency Identity Protocols Class 1 Generation-2 UHF RFID Air Interface Protocol for Communications at 860 MHz–690 Mhz.

[26] Cha J.R., Kim J.H. (2005). Novel anti-collision algorithms for fast object identification in RFID system. Proceedings of the 11th International Conference on Parallel and Distributed Systems.

[27] Klair D.K., Wu Chin K., Raad R. On the Accuracy of RFID Tag Estimation Functions. Proceedings of the International Symposium on Communications and Information Technologies (ISCIT).

[28] Schoute F.C. (1983). Dynamic Frame Length ALOHA. IEEE Trans. Commun..

[29] Bueno-Delgado M.V., Vales-Alonso J., González-Castaño F. Analysis of DFSA anti-collision protocols in passive RFID enviroments. Proceedings of the IEEE International Conference of the Industrial Electronics Society (IECON).

[30] Verblunsky S. (1949). On the Least Number of Unit Circles Which Can Cover a Square. J. Lond. Math. Soc..

[31] Friedman E. Disk Covering Problem. http://www2.stetson.edu/$\sim$efriedma/circovcir/.

[32] Bonuccelli M.A., Lonetti F., Martelli F. (2007). Instant collision resolution for tag identification in RFID networks. Ad Hoc Netw..

[33] Lee S.R., Joo S.D., Lee C.W. An enhanced dynamic framed slotted ALOHA algorithm for RFID tag identification. Proceedings of the 2nd International Annual Conference on Mobile and Ubiquitous Systems: Networking and Services.

[34] Bueno-Delgado M.V., Ferrero R., Gandino F., Pavon-Marino P., Rebaudengo M. (2013). A Geometric Distribution Reader Anti-Collision Protocol for RFID Dense Reader Environments. IEEE Trans. Autom. Sci. Eng..

[35] Farahani S. (2008). Zigbee Wireless Networks and Transceivers.

